# High-throughput label-free cell detection and counting from diffraction patterns with deep fully convolutional neural networks

**DOI:** 10.1117/1.JBO.26.3.036001

**Published:** 2021-03-08

**Authors:** Faliu Yi, Seonghwan Park, Inkyu Moon

**Affiliations:** a University of Texas Southwestern Medical Center, Department of Clinical Science, Dallas, Texas, United States; b Daegu Gyeongbuk Institute of Science and Technology, Department of Robotics Engineering, Dalseong-gun, Daegu, Republic of Korea

**Keywords:** cell counting, deep learning, digital holographic microscopy, holography application, optical information processing, red blood cell analysis

## Abstract

**Significance:** Digital holographic microscopy (DHM) is a promising technique for the study of semitransparent biological specimen such as red blood cells (RBCs). It is important and meaningful to detect and count biological cells at the single cell level in biomedical images for biomarker discovery and disease diagnostics. However, the biological cell analysis based on phase information of images is inefficient due to the complexity of numerical phase reconstruction algorithm applied to raw hologram images. New cell study methods based on diffraction pattern directly are desirable.

**Aim:** Deep fully convolutional networks (FCNs) were developed on raw hologram images directly for high-throughput label-free cell detection and counting to assist the biological cell analysis in the future.

**Approach:** The raw diffraction patterns of RBCs were recorded by use of DHM. Ground-truth mask images were labeled based on phase images reconstructed from RBC holograms using numerical reconstruction algorithm. A deep FCN, which is UNet, was trained on the diffraction pattern images to achieve the label-free cell detection and counting.

**Results:** The implemented deep FCNs provide a promising way to high-throughput and label-free counting of RBCs with a counting accuracy of 99% at a throughput rate of greater than 288 cells per second and 200  μm×200  μm field of view at the single cell level. Compared to convolutional neural networks, the FCNs can get much better results in terms of accuracy and throughput rate.

**Conclusions:** High-throughput label-free cell detection and counting were successfully achieved from diffraction patterns with deep FCNs. It is a promising approach for biological specimen analysis based on raw hologram directly.

## 1 Introduction

Label-free quantitative phase imaging techniques, such as digital holographic microscopy (DHM), have been widely researched and applied in biomedical area.^1^^–^^8^ Especially, the DHM system has promising application values for the observation and study of transparent or semitransparent biological specimens, such as blood cells and cardiomyocytes, because two-dimensional (2D) imaging systems only record the intensity values of biological samples and lose a lot of three-dimensional (3D) information that is important for the further analysis.^1^^,^^9^^–^^12^ Conventionally, the biological specimens are visualized by DHM and the diffraction patterns between the object wave and reference wave are recoded in a CMOS or charge-coupled device (CCD). Afterward, the numerical reconstruction method is used to reconstruct the phase image from the diffraction pattern and the multiple targets within the reconstructed phase images are quantitatively analyzed.^13^^–^^22^ Therefore, the numerical reconstruction algorithm is usually inevitable for the target analysis.

In Refs. 14–16, 18, and 20, the phase images of multiple red blood cells (RBCs) are reconstructed from holograms recorded using DHM system. Then, some digital segmentation algorithms are applied to the reconstructed phase images to extract the target cells that are used for the quantitative analysis, such as cell feature measurement, cell distribution comparison, and time-lapse feature observation. In Refs. 4 and 23, the phase images of cardiomyocytes are numerically reconstructed from the diffraction patterns recorded by DHM and the beating profiles of different cardiomyocytes are researched. There are also many other works related to analysis of transparent or semitransparent biological specimen or even non-biological targets that are based on the phase images reconstructed from DHM-recorded hologram.^23^^–^^27^ Nevertheless, it is obvious that the numerical reconstruction steps have to be conducted before starting the research of the multiple targets within the imaging sample. However, the numerical reconstruction algorithms, which may consist of processes such as spatial filtering, phase unwrapping, or numerical propagations of complex diffraction wave potentially, make the final analysis complicated and inefficient.^28^ Therefore, the multiple target analysis can definitely benefit from the novel algorithm that can reduce the complexity of numerical reconstruction step or totally avoid the step of numerical reconstruction.

The RBCs contain hemoglobin that carries oxygen to the body’s tissue and the number of RBCs we have can affect how much oxygen our tissues receive while the tissues need oxygen to function. The patient would experience symptoms and complications with a low or high number of RBCs.^29^ Therefore, the RBC detection would be helpful to the RBC classification so that benefits to the analysis of blood-related disease.^30^ The detection of RBC also benefits the quantitative analysis of cells that may determine the quality of the blood.^14^ There are different kinds of image processing algorithms for the multiple cell detection and counting and these images are either from 2D imaging techniques such as bright-field microscopy or phase images that are obtained by DHM.^1^^,^^12^ The 2D imaging techniques cannot provide enough information such as depth feature for the analysis of semitransparent or transparent biological specimen while the holographic imaging schemes can deliver sufficient features for the imaging target samples.^3^^,^^12^^,^^18^ Therefore, the holographic imaging systems are widely used for the visualization and study of biological semitransparent or transparent samples. There are also many research studies regarding the biological specimen or flow cytometry using the DHM.^31^^,^^32^ However, the studies are based on the phase images that need the help of numerical reconstruction algorithms. The understandable reason is that the phase image can reveal the imaging target better while the target in the raw hologram is much vague. The edge of the cell in the diffraction pattern recorded in DHM is usually not well defined while that in the phase image is much clearer. Therefore, it may be difficult to conduct the cell analysis based on the raw hologram using traditional image processing methods due to the ambiguity of the biological targets in the diffraction pattern. Fortunately, the diffraction pattern recorded by the DHM can be studied using the deep neural networks because the deep neural networks do not need the researcher to manually define the specific features within the hologram image and can automatically extract useful features by itself according to the definition of final goal in the networks. Researchers have also successfully introduced deep learning scheme into 3D imaging field.^28^^,^^33^^–^^36^ In Ref. 28, the authors have demonstrated that a deep convolutional neural network (CNN) can be applied to the raw hologram directly for the detection and counting of imaging targets. However, the method in Ref. 28 needs to define the size of image patch for training the CNN algorithm and it will be difficult to find a good patch size for the improved performance. In addition, prediction based on the small image patch where each patch represents one pixel will be time consuming and inefficient when the input is the hologram image because a hologram has a lot of pixel and there are a lot of overlap among different image patches. On the other hand, the fully convolutional network (FCN) algorithm^37^ can avoid the drawbacks that exist in CNN for the target detection, segmentation, and counting within images.

In this paper, we propose to use one of the FCN techniques, which is UNet algorithm,^38^ for the cell detection and counting in the diffraction pattern recorded in DHM. The UNet algorithm is an end-to-end segmentation method where both the input and output are images and have the same size. Therefore, the UNet algorithm can avoid the disadvantages such as time intensiveness and difficulty in patch size choice which existed in the CNN algorithm for pixel-based image segmentation. In addition, the UNet algorithm is invariant to the size of the input image while the CNN algorithm needs the input of the image to have a fixed size. After the UNet algorithm is trained, the multiple cell targets within diffraction pattern are directly analyzed without any numerical reconstruction processing. Similar to UNet, other FCNs, such as Pyramid Scene Parsing Network (PSPNet)^39^ and Deeplab V3+ (encoder–decoder with atrous separable convolution for semantic image segmentation),^40^ may also work for the multiple cell detection and counting in hologram images. Different from works in Refs. 41 and 42, which present methods to process raw image from 2D imaging microcopy, we only focus on the processing of raw hologram from the DHM. The remainder of this paper is organized as follows. Section 2 presents the materials and method. Section 3 shows experimental results. The discussion and conclusion are included in Sec. 4.

## 2 Materials and Method

### 2.1 Digital Holographic Microscopy

**Fig. 1:**
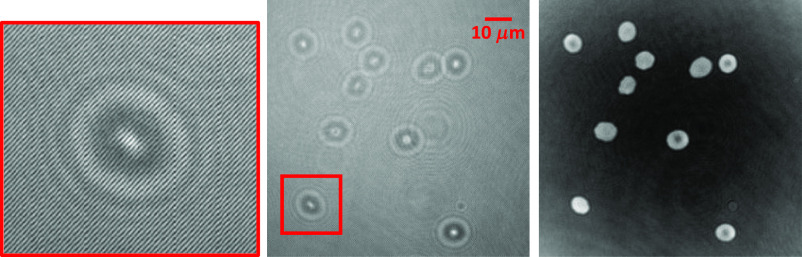
Hologram and phase image. Left: digital hologram of RBCs recorded in DHM. Right: the RBCs’ phase image reconstructed from the digital hologram.

In this section, we briefly explain the basic concepts of the label-free off-axis DHM^12^^,^^16^^,^^43^^,^^44^ to record the diffraction pattern of biological cells. In our experiment, a λ=682-nm laser diode source is used as the light source for the illumination of target samples. Laser light is divided into an object wave and a reference wave. The object wave illuminates the samples and the microscope objective (MO) collects and magnifies the wavefront of the object. The angle θ between the interfering waves in the off-axis DHM system can be experimentally tuned and fixed. Then, a hologram between the enlarged diffraction pattern and the reference wave is captured via a CCD. A quantitative phase image is finally obtained by numerical calculation of the recorded off-axis holograms.^43^^,^^44^ In our DHM setup, the MO inserted in the object wave arm introduces phase aberration, but it can be numerically resolved by multiplying the reconstructed wavefront with the complex conjugate of the phase aberration.^43^^,^^44^ Figure 1 shows one off-axis hologram of RBCs and its corresponding reconstructed phase images of RBCs.

### 2.2 Sample Preparation

Blood sample was drawn from healthy male donors using a syringe. The collected blood was diluted at a ratio of 100:14 (v/v) in CPDA-1. RBCs were sedimented by centrifuging at 200 g for 5 min, and then the buffer coat was gently removed. The RBCs were mixed with HEPA buffer at 0.2% hematocrit, and 1 ml of the erythrocyte suspension was diluted to 15 ml using HEPA buffer. RBCs diluted with HEPA buffer were dropped onto imaging slides. The slide and chamber were mounted on the DHM and stabilized for 5 min at room temperature. The blood was obtained through legitimate procedures in accordance with the guidelines and regulations (DGIST-180713-BR-012-01) approved by the bio-safety committee, DGIST, and institutional review boards in Korea. The experiments were finished within a few hours after sample collection, and the blood was disposed.

### 2.3 UNet Algorithm

**Fig. 2:**
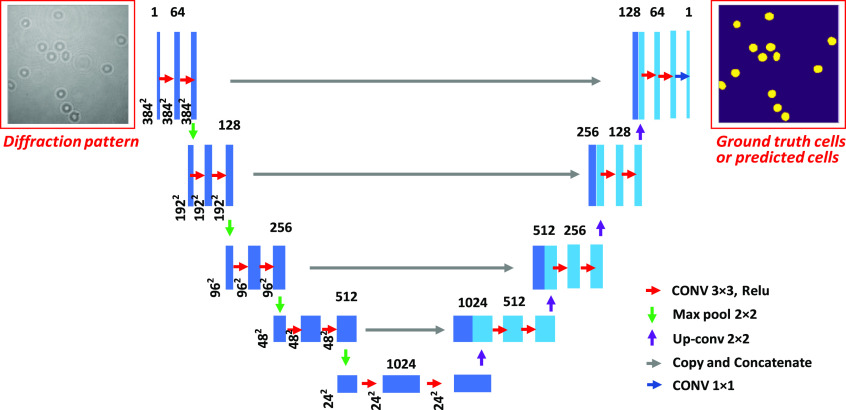
UNet structure for the multiple cell detection and counting in diffraction pattern.

The UNet model is one kind of deep FCN. There are no fully connected layers in the UNet and thus it can handle an image with different sizes. The UNet structure we used for the multiple cell detection and counting is shown in Fig. 2. We may refer to the specific architecture in Fig. 2 when we mention the used UNet model in this study. Basically, there are convolution, rectified linear unit (Relu) activation, up-convolution, max pooling, and concatenate layer in our used UNet structure.^38^ The convolution layer is for feature extraction from the input image. It is a mathematical operation between two inputs such as image or feature maps and a kernel that consists of learning parameters. The convolution operation can preserve the spatial relationship among pixels and learns the image features based on small squares of input image or feature map.^45^ The Relu activation is defined as y=max⁡(0,x), where x is the input feature value, y is the output, and max() is the maximum operation function. The Relu activation function is used to introduce the non-linearity into the networks. Compared with sigmoid activation function within the deep neural networks, the Relu activation function can reduce the gradient vanish problem during algorithm training.^45^ The up-convolution layer is usually used in the encoder–decoder architecture networks such as FCN and UNet algorithm. The up-convolution operation reconstructs the spatial resolution from before and then performs a convolution operation. Therefore, it can be implemented with the combination of upscaling of an image and a convolution layer. The up-convolution is also refered to as deconvolution layer or transposed convolution layer in different research papers.^45^^,^^46^ Max pooling layer extracts the largest element from the rectified feature map and it is one kind of pooling operations.^45^^,^^46^ Pooling layer in deep FCNs can reduce the dimensionality of each feature map and benefits to the network’s computation. Even though the size of feature map is reduced in max pooling layer, the important feature information can still be retained. To some extent, the max pooling layer in deep FCNs can also make the algorithm robust to the translation of target within the input images. The concatenate layer is a utility layer that merges its multiple input feature maps to one group of feature map.

In Fig. 2, the single number, such as 64 and 128, means the number of feature map in that layer while the square number, such as $384^{2}$, represents the spatial size of the feature map. In our UNet algorithm, the diffraction pattern from DHM is used as the input image and the size used for feeding the UNet is $384^{2}$, which can be resized from the original diffraction pattern. The ground truth image is a binary image where the foreground denotes the target cells. The sigmoid function is applied to the last layer of UNet so as to make the value of each pixel in output image between 0 and 1. The loss function is defined between the UNet predicted output and the ground truth image. Here, the combination of soft Dice coefficient and cross-entropy loss (logarithmic loss for the two classes case) L, which is defined as the following equation is used to learn the parameters in UNet architecture based on back-propagation algorithm:^45^^,^^46^

$$L=(1-\frac{\overset{N}{\underset{i=1}{\sum }}2(y_{i}{\overset{˜}{y}}_{i})}{\overset{N}{\underset{i=1}{\sum }}(y_{i}+{\overset{˜}{y}}_{i})})-\frac{1}{N}\overset{N}{\underset{i=1}{\sum }}[y_{i}\;\log({\overset{˜}{y}}_{i})+(1-y_{i})\log(1-{\overset{˜}{y}}_{i})],$$ (1)

where N is the total number of pixels in the output map, $y_{i}$ is the true category of i’th pixel in the ground truth image, and ${\overset{˜}{y}}_{i}$ is the probability of belonging to the foreground category for the i’th pixel in the output map from UNet algorithm.

### 2.4 Procedure of Multiple Cell Detection and Counting

There are two main steps for the multiple cell detection and counting in diffraction pattern obtained from DHM, that is, steps of learning and prediction. In the learning stage, the input is the raw hologram image and the output is a probability map. The output is then compared with the ground truth image and the parameters in UNet are learned based on the cross-entropy loss function using back-propagation algorithm. After the UNet is trained, the networks’ output with a threshold applied is viewed as the prediction results in the prediction phase for a new input diffraction pattern. The threshold value is set to be 0.5, which means the pixel is categorized into cells when the probability value is bigger than 0.5. Otherwise, the corresponding pixel is labeled as background. Before the learning starts, the parameters of the UNet in the encoder part are initialized with a pre-trained VGG networks^45^^,^^47^ while the first layer and other layers in decoder part are initialized using “He initialization” method.^48^ During the training, the input images are randomly augmented so as to increase the training dataset and reduce the over-fitting problem. Some augmentation methods are described in the data preparation section.

**Fig. 3:**
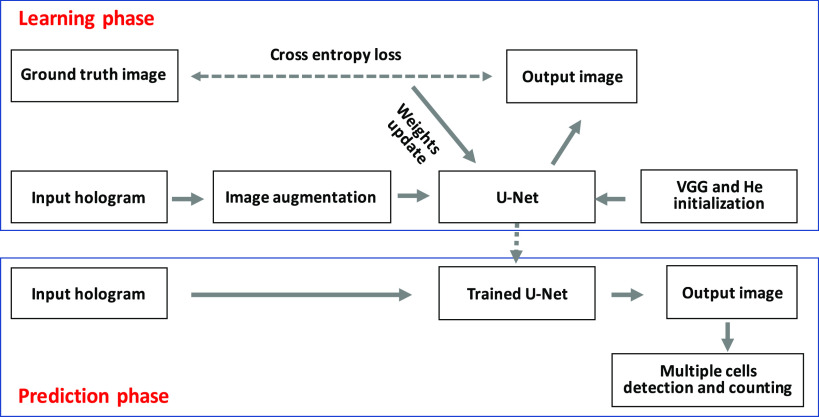
Procedure of multiple cell detection and counting at the single cell level using UNet.

The multiple cells are segmented from the UNet algorithm and we obtain a binary image where foreground represents the cells. Before counting the multiple cells, the binary mask image is processed using morphological opening operation where the structuring element is a disk with radius of 3.^49^ This morphological operation can remove some small isolated targets, which are probably from noise and smooth the target edge at the same time. Moreover, we have excluded these cells on the frame boundary whose area is smaller than 40 pixels from both ground truth and predicted image for the cell counting. Then, the object in the processed binary image is labeled. That is, a unique label is assigned to all pixels of each object in the binary image. This can be easily done using connected-component labeling algorithm.^49^ After labeling, the maximum value in the labeling image is the number of cells in the detected image. It may be also a good idea to take the multiple cell counting processing as a loss layer and achieve the cell detection and counting from the network directly. We only use the post-processing method to automatically count multiple cells from algorithm output in this paper. Figure 3 gives the procedure of multiple cell detection and counting using UNet algorithm.

### 2.5 Data Preparation

**Fig. 4:**
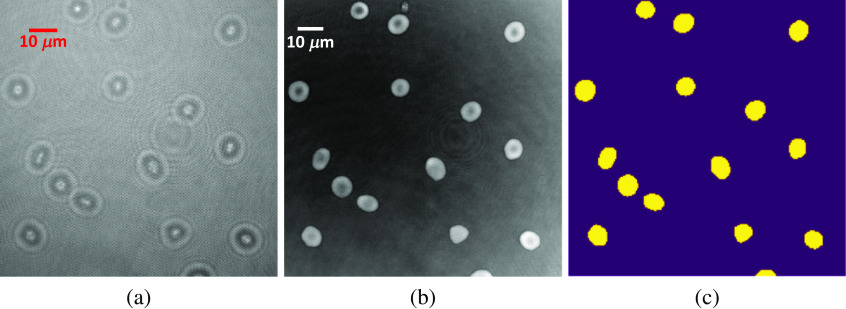
Illustration of hologram, phase, and labeled images. (a) Hologram image, (b) reconstructed phase image, and (c) labeled ground truth image.

The holograms of the RBCs from a healthy donor are acquired from off-axis DHM. Even though our algorithm is based on the raw diffraction pattern and the prediction does not need the reconstructed phase images, we still reconstruct the phase images from the holograms for the purpose of UNet training and evaluation. The numerical reconstruction algorithm in Ref. 44 is used for the phase image reconstruction where the size of the phase image is 700×700  pixels, which corresponds to the 100  μm×100  μm field of view (FOV). The biologist has manually labeled 150 images for our algorithm development. The labeling processing is based on the reconstructed phase images. The illustration of RBCs hologram, RBCs phase image, and the labeled image (ground truth) are given in Fig. 4.

Since there are some hyper-parameters in the deep learning algorithm training such as learning rate and weigh decay, we divide our dataset into two groups that are training and validation dataset for the purpose of hyper-parameter tuning and algorithm evaluation. 125 hologram images are used as training and 25 hologram images are taken as validation dataset. For the performance evaluation, the 5-fold cross-validation method based on the training dataset is conducted.

All of the 150 holograms are obtained from DHM with 40× magnification. We also have 50 hologram images that include correspondingly labeled ground truth images where these images are acquired from DHM under 20× magnification and 70 bead hologram images that are captured under 40× magnification. The original size of diffraction pattern from DHM under 20× magnification is 700×700  pixels and the corresponding FOV is 200  μm×200  μm while they are 782×782  pixels and 200  μm×200  μm, respectively, for the bead hologram. All the hologram images have only one channel and it is also the input channel for the deep learning algorithms used in this paper. We will use these data to test the generalization of the UNet algorithm and compare the prediction results under training dataset with different magnifications.

**Fig. 5:**
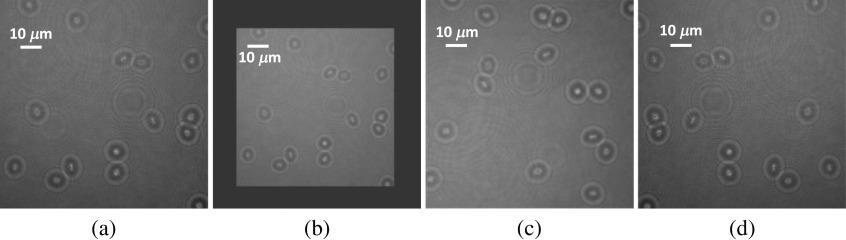
Illustration of image augmentation. (a) Original image, (b) image resizing, (c) image rotation, and (d) image flipping.

The image normalization and augmentation are applied to each image before feeding it to the UNet algorithm. The image normalization is conducted by subtracting the mean value and then dividing by the standard deviation where the mean and standard deviation are calculated from the training dataset. The image augmentation we used during algorithm training consists of image cropping, resizing, translation, rotation, and flipping. These augmentation methods are from a python package called “imgaug.”^50^ Some of the image augmentation examples are given in Fig. 5. The image is padded with zero value after resizing when it is smaller than 384×384, which is the input size to the UNet algorithm.

### 2.6 Hardware and Hyper-Parameter Configuration

The UNet algorithm is trained on a server that has 48 CPUs [Intel(R) Xeon(R) CPU E5-2650], one P100 Nvidia GPU, and Ubuntu 16.04 operating system. Both the training and prediction are based on GPU parallel computing and the algorithm is implemented based on “Pytorch” deep learning framework.^51^ The batch size used in the learning phase is 4 and an optimized gradient descent algorithm called momentum is adopted as the optimization method to optimize the loss function, which is the combination of soft Dice coefficient and cross-entropy loss, and the momentum value is set to be 0.9. The learning rate is initialized as 0.01 and decreased by a factor of 10 every 30 epochs. L2 regularization is used to reduce the over-fitting problem and the value is set to be 0.0001. The number of epochs is initialized to be 60.

### 2.7 Evaluation Metrics

There are several metrics used to evaluate the multiple cell detection and counting. First, we adopt the metrics accuracy, sensitivity, and Dice score coefficient (DSC). Sensitivity measures the proportion of actual positive cases that correctly identified as such. A high sensitivity rarely overlooks an actual positive. The sensitivity within a predicted image is given as

$$\text{Sensitivity}=\frac{\mathrm{TP}}{\mathrm{TP}+\mathrm{FN}},$$ (2)

where TP is the true positive and FN is the false negative pixels within image. The metric accuracy is the evaluation of all pixels within the image and defined as follows:

$$\text{Accuracy}=\frac{\mathrm{TP}+\mathrm{TN}}{\mathrm{TP}+\mathrm{TN}+\mathrm{FP}+\mathrm{FN}},$$ (3)

where true negative (TN) is the number of non-cells pixels that are correctly classified and FP is the false positive. Accuracy is the percentage of correctly classified pixels, including both cells and background, out of the total number of pixels. DSC is defined as follows:

$$\mathrm{DSC}=\frac{2\mathrm{TP}}{2\mathrm{TP}+\mathrm{FP}+\mathrm{FN}},$$ (4)

and it is also used to evaluate the multiple cell segmentation results. Another two metrics, which are Hausdorff distance (HD) and 95th percentile Hausdorff distance (95th HD), are also included for the performance evaluation.^52^^,^^53^ They are computed between the binary objects in two images, which are ground truth and predicted image. It is an indicator of the largest segmentation error. Here, HD is defined as the maximum surface distance between the objects. That is, HD is measured between boundaries of the predicted and ground-truth segmentation. For two point sets X and Y (see Fig. S1 in the Supplementary Material), the HD(X,Y) is the longest distance one has to travel from a point in one of the two sets to its closest point in the other set. It can be estimated as follows:

HD(X,Y)=max(hd(X,Y),s(Y,X)), (5)

where

$$\mathrm{hd}(X,Y)=\underset{x\in X}{\max}\;\underset{y\in Y}{\min}{\left\|x-y\right\|}_{2},$$ (6)

$$\mathrm{hd}(Y,X)=\underset{y\in Y}{\max}\;\underset{x\in X}{\min}{\left\|x-y\right\|}_{2},$$ (7)

and ${\left\|x-y\right\|}_{2}$ is the Euclidean distance between point x and y. Compared to the HD, the 95th HD is slightly more stable to small outliers and is commonly used in image segmentation. For both HD and 95th HD, the value is the smaller the better. For more details about HD and 95th HD, refer to Refs. 52 and 53. We also define the correctly counted cell and over-counted cells metrics for the cell counting evaluation. A cell is counted as correctly counted cell if the centroid point of an isolated region in the predicted image from UNet is within the cell of ground truth image. Otherwise, it is counted as over-counted cell. In case of more than two isolated regions’ centroid points are located at the same cell in the ground truth image, only one predicted isolated region is regarded as correctly counted cell and the other regions are counted as over-counted cells (see Fig. S2 in the Supplementary Material). In addition, the throughput rate, which is the counted number of cells per second, is also given. That is, the unit of throughput rate is cells/s and it is the higher the better. All these metrics are measured based on the 5-fold cross-validation technique.

## 3 Experimental Results

The UNet algorithm is trained using the 5-fold cross-validation scheme based on training data and the hyper-parameters are tuned based on the validation dataset. Here, the DSC as defined in Eq. (4) is used to evaluate the UNet algorithm and tune the hyper-parameters. Here, the hyper-parameters we try to tune are learning rate and weight decay. We use the grid search method to tune the algorithm. The candidate values for learning rate and weight decay during grid search are these empirical values. The varied values of learning rate and weight decay, and their corresponding best DSC values on validation dataset during UNet learning are shown in Table 1.

**Table 1:** DSC value from varied learning rate and weight decay.

Weight decay	Learning rate			
	0.01	0.001	0.0001	0.00001
0.01	0.9112	0.8788	0.8376	0.4053
0.001	0.9303	0.8626	0.8432	0.4244
0.0001	**0.9321**	0.8542	0.8468	0.3648
0.00001	0.9190	0.8800	0.8439	0.3325

**Fig. 6:**
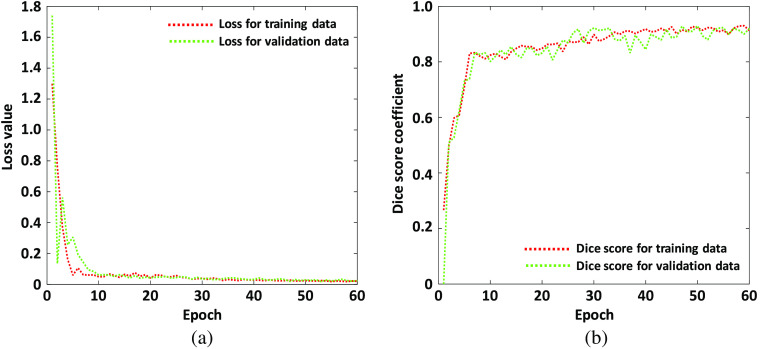
Loss and Dice score value during algorithm training: (a) loss values for training and validation dataset and (b) Dice score values for training and validation dataset.

It is noted from Table 1 that the UNet algorithm get the best Dice score value 0.9321 (highlighted in bold) on validation dataset when the learning rate is 0.01 and the weight decay value is 0.0001. Therefore, the final UNet model is trained based on learning rate and weight decay with values of 0.01 and 0.0001, respectively. We have to mention here that the 5-fold cross validation is used for the performance evaluation and the hyper-parameters are tuned in the first round of 5-fold cross validation while the other four rounds use the tuned hyper-parameters in the first round. Figure 6 shows the learning curves between epoch number versus loss value and DSC value. It is indicted in Fig. 6 that best DSC value is achieved when the epoch number is 50. Therefore, the final model we used is from the trained parameters at epoch 50. The average execution time for the UNet training with epoch 50 is 633.2311 s and the prediction time for a 384×384 image is 0.1050 s. The computation time for both the UNet training and prediction is very promising.

**Fig. 7:**
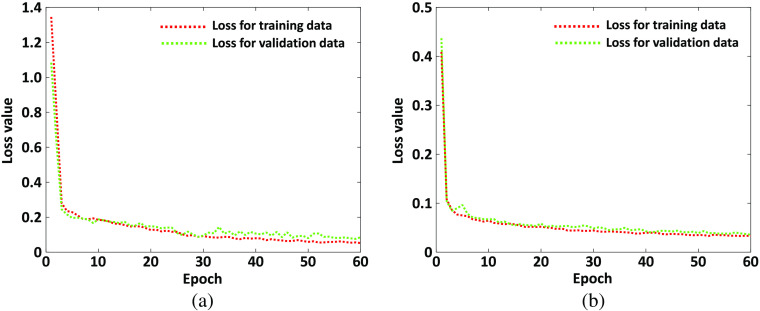
Loss values during algorithm training for (a) PSPNet and (b) CNN algorithm.

Once the hyper-parameters are tuned and the final UNet is trained, the algorithm is then evaluated based on the 5-fold cross-validation technique. Up to now, all of the hologram images we used for the algorithm training are obtained from DHM under 40× magnification. To test the performance of the trained algorithm on hologram images obtained from different condition, we also evaluate the trained UNet algorithm on hologram images obtained from DHM under 20× magnification and bead hologram images under 40× magnification. Table 2 shows some of the metric values for hologram images obtained from 40× magnifications (125 RBC hologram images in 5 folds) and 20× magnifications (50 RBC hologram images in 5 folds), and bead hologram images (70 bead hologram images in 5 folds). The other metric values that are not included in Table 2 are given in Table S1 in the Supplementary Material. The average and standard deviation value from the 5 folds are provided in Table 2. Here, the total number of cells is the number of cells in the ground truth images and it is the summation of correctly counted cells and the missing counted cells. To compare the performance of different methods, the CNN algorithm present in Ref. 28 for the multiple cell counting from hologram images is adopted. Moreover, another famous FCNs, which is PSPNet, is also included for the performance evaluation. Here, all hyper-parameters of the algorithms are tuned using the validation dataset. The loss values for both CNN and PSPNet algorithms on one training and validation dataset are present in Fig. 7. The initial learning rate for the CNN is 0.01 and it is 0.001 for the PSPNet. The epoch number of CNN and PSPNet is 60. The weight decay value for both CNN and PSPNet is 0.0001. The input image size for CNN algorithm is 80×80 and the architecture is the same as that in Ref. 28 (see Fig. S3 in the Supplementary Material). The mirroring method is used for the patch extraction along the image border. The used PSPNet architecture is given in Fig. S4 in the Supplementary Material. The input image size for PSPNet is 384×384 and the Resnet34^54^ is used as the encoder, which is CNN part shown in Fig. S4 in the Supplementary Material. All configurations about POOL, CONV, and UPSAMPLE can be found in Ref. 39. Specific architecture is defined in Figs S3 and S4 in the Supplementary Material, as a reference for the CNN and PSPNet algorithm used in this paper. Results from CNN and PSPNet algorithms are also included in Table 2. The average training time for CNN and PSPNet are 8819.83 and 576.8960 s, respectively. The average prediction time for a 384×384 image is 36.8764 and 0.0872 s for the CNN and PSPNet algorithm, respectively. It meets the expectation that the FCN-based algorithm, such as UNet and PSPNet, can achieve much faster prediction time than CNN algorithm for semantic segmentation because the FCN method can automatically finish all pixel predictions in parallel while each pixel that is denoted with a 80×80 image batch within image is sequentially predicted in CNN algorithm in a sliding window scheme. The throughput rates for CNN and PSPNet algorithm for different dataset are also given in Table 2.

**Table 2:** Evaluation of model trained on hologram images under 40× magnification.

Metrics	RBC hologram (40×) (mean/std)			RBC hologram (20×) (mean/std)			Bead hologram (40×) (mean/std)		
	CNN	UNet	PSPNet	CNN	UNet	PSPNet	CNN	UNet	PSPNet
DSC	0.85/0.003	0.91/0.002	0.88/0.003	0.32/0.009	0.42/0.02	0.35/0.01	0.21/0.007	0.26/0.01	0.31/0.01
HD	20.90/6.25	14.88/5.51	17.37/4.35	50.48/3.36	43.55/6.93	35.74/7.42	105.17/11.13	79.55/7.4	105.8/5.9
Sensitivity	0.92/0.006	0.96/0.003	0.92/0.003	0.81/0.01	0.83/0.02	0.84/0.02	0.51/0.03	0.76/0.04	0.46/0.04
Accuracy	0.97/0.0004	0.99/0.0003	0.98/0.0005	0.90/0.003	0.92/0.003	0.94/0.002	0.93/0.0004	0.95/0.001	0.95/0.0006
Throughput rate	0.37/0.01	125.4/4.65	148.71/6.6	1.04/0.08	430/27.14	405.73/33.69	0.93/0.04	422.47/47.05	305.96/22.83

**Fig. 8:**
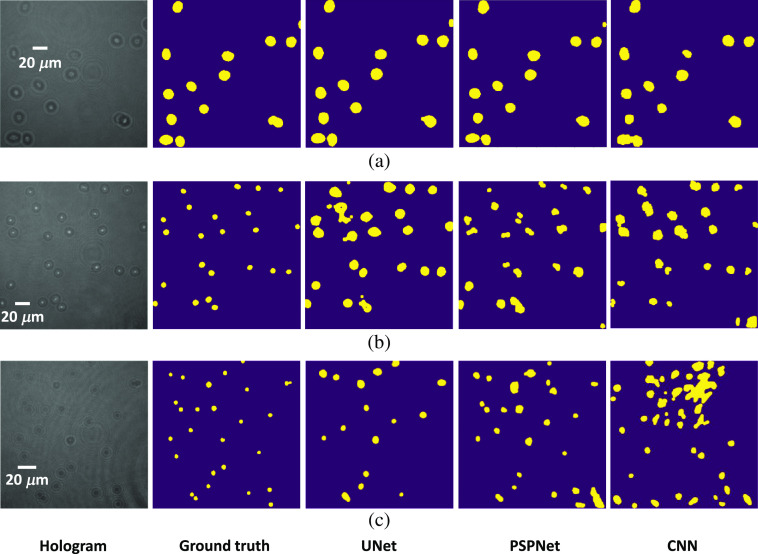
Illustration of prediction results. (a) 40× magnification image, (b) 20× magnification image, and (c) bead hologram image where UNet, PSPNet, and CNN algorithm are trained on 40× magnification images.

It can be found from Table 2 that the trained model performs very well for the hologram images that are obtained from DHM under 40× magnification where these images are similar as these used in the UNet training. However, it looks that the UNet algorithm trained from hologram images under 40× magnification cannot get good prediction results for hologram images obtained from DHM under 20× magnification and bead hologram images because the DSC value is low and the number of over-counted cell is high. That is, the algorithm’s performance is decreased when the testing dataset is very different from the training dataset. It is also revealed from Table 2 that the UNet and PSPNet algorithms get very similar results and both of their results are better than that from CNN algorithm. Figure 8 shows some of the prediction results from algorithm CNN, UNet, and PSPNet for hologram images obtained from DHM under 40× and 20× magnification, and bead hologram images, respectively.

**Fig. 9:**
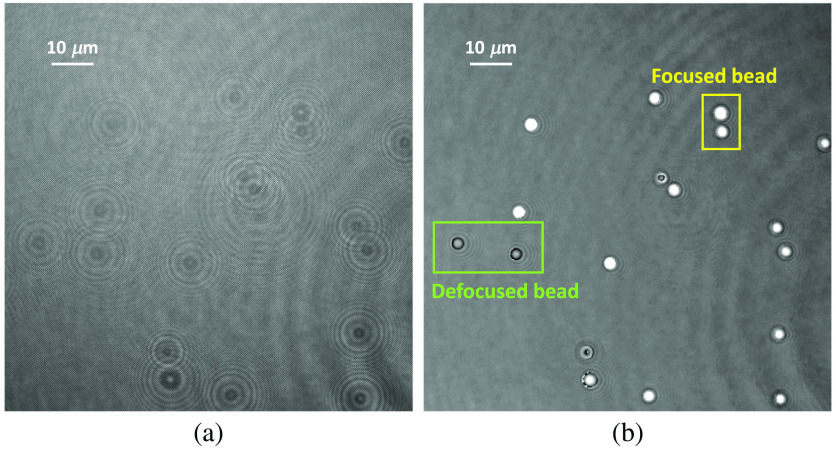
Illustration of focused and defocused beads in the bead hologram images. (a) Bead hologram image and (b) corresponding reconstructed phase image with some focused and defocused beads indicated.

To investigate the algorithm more, we re-trained these algorithms. However, we added 50 hologram images obtained from DHM under 20× magnification and 70 bead hologram images under 40× magnification into the training dataset this time while the 5-fold cross validation is still used. The hyper-parameter is tuned the same way as we did previously, which is conducted based on the validation dataset. Table 3 shows some of the evaluation results obtained from DHM system under 40× and 20× magnification, and bead hologram images based on the 5-fold cross validation. The other metric values that are not included in Table 3 are given in Table S2 in the Supplementary Material. Compared results in Table 3 with these in Table 2, we can find that the algorithm performance on hologram images obtained from DHM under 20× magnification and bead hologram images is greatly increased while the performance on hologram images obtained from 40× magnification is still very good. It is also noted that the UNet performance on hologram images under 20× magnification is worse than these images under 40× magnification. The reason may be the deficiency of hologram images of 20× magnification in the training dataset because there are 125 hologram images with 40× magnification and only 50 hologram images with 20× magnification in the training dataset. The prediction performance for hologram images with 40× magnification is also better than that of bead hologram images. One reason may be the deficiency of bead hologram images (70 bead hologram images) used in the training and the other reason may be the complexity of target in the bead hologram images because the bead hologram image includes both focused and defocused objects. In other words, some beads are located at a different focus distance to the other beads. These focused and defocused beads are illustrated in the reconstructed phase images in Fig. 9.

**Table 3:** Evaluation of model trained on hologram images from different source.

Metrics	RBC hologram (40×) (mean/std)			RBC hologram (20×) (mean/std)			Bead hologram (40×) (mean/std)		
	CNN	UNet	PSPNet	CNN	UNet	PSPNet	CNN	UNet	PSPNet
DSC	0.86/0.003	0.91/0.002	0.88/0.003	0.76/0.01	0.88/0.01	0.88/0.02	0.57/0.03	0.72/0.02	0.70/0.02
HD	17.85/4.27	13.17/4.32	15.44/3.85	25.85/9.43	23.08/8.96	22.32/8.17	78.76/6.4	43.51/6.81	51.09/9.38
Sensitivity	0.90/0.004	0.95/0.004	0.93/0.005	0.85/0.01	0.90/0.02	0.93/0.01	0.48/0.03	0.72/0.03	0.68/0.03
Accuracy	0.97/0.0003	0.99/0.0003	0.98/0.0007	0.96/0.0005	0.98/0.0004	0.98/0.001	0.94/0.0004	0.98/0.0003	0.95/0.0003
Throughput rate	0.36/0.01	125.18/4.42	149.90/6.06	0.77/0.08	288.41/26.9	355.73/35.37	0.50/0.04	221.58/17.42	267.01/17.21

**Fig. 10:**
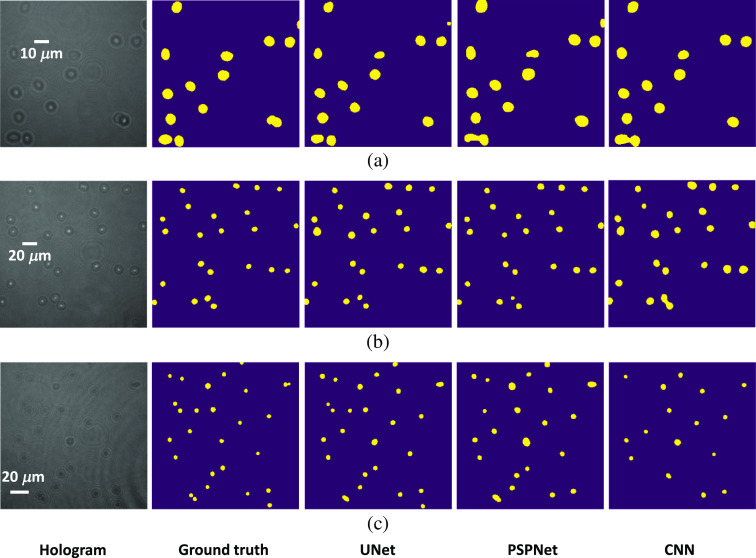
Illustration of prediction results. (a) 40× magnification image, (b) 20× magnification image, and (c) bead hologram image where UNet, PSPNet, and CNN algorithm are trained on 40× and 20× magnification images.

Figures 10 also shows the prediction results for input hologram images with 40× and 20× magnification, and bead hologram images for CNN, UNet, and PSPNet algorithm. It is found that the prediction results in Figs. 8(a) and 10(a) are very similar, and the results in Figs. 10(b) and 10(c) are much better than results in Figs. 8(b) and 8(c). Moreover, both the focused and defocused beads in the bead hologram images are detected when the algorithm is trained on hologram images obtained in different source. When it comes to the algorithm, both UNet and PSPNet get better results than CNN. These results also imply that it is better to train a deep learning algorithm using training dataset that should be similar with these in the testing space. That is, the distribution of training data and testing data should be similar, and this is also the essence of all machine learning algorithms. In a word, these simulation results have verified that the deep FCNs can be used to detect and count the multiple cells from the hologram images directly, and it is not necessary to apply the numerical reconstruction algorithm for the phase image reconstruction. Therefore, the proposed algorithm can improve the efficiency of label-free detection and counting of multiple cells at the single cell level. Additionally, our proposed high-throughput cell counting schemes at the single cell level may be further enhanced by increasing the image FOV. This would lead to future studies to see if deep FCNs may detect, count, and classify biological cells in lower resolution hologram images.

**Fig. 11:**
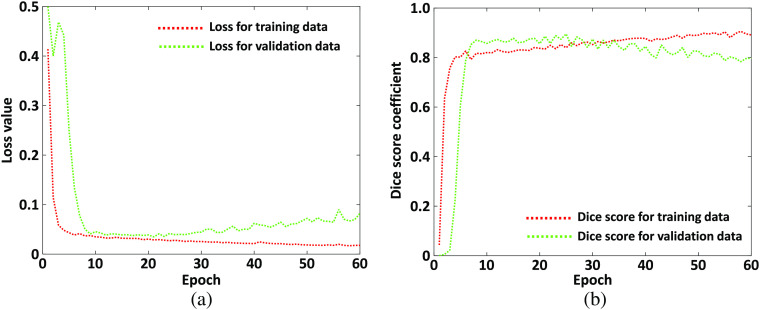
UNet learning curve between (a) epoch number versus loss value and (b) DSC value without image augmentation.

The multiple cell detection and counting from diffraction patterns with deep fully convolutional neural can avoid the numerical phase reconstruction algorithm, which is complex and inefficient. However, it is expected that the results from diffraction pattern are comparable with these from the reconstructed phase image. Consequently, we have tested the UNet algorithm based on phase images reconstructed from holograms that are obtained from DHM under 40× magnification. The training process is the same as the one we conducted using the diffraction pattern images. Table 4 gives the 5-fold cross-validation evaluation results for the UNet algorithm trained on reconstructed phase images. It can be seen from Table 4 that the results for UNet trained on reconstructed phase image and diffraction patterns are very similar. That is, multiple cell detection and counting from diffraction patterns is feasible. In our algorithm training, we have applied image augmentation approach to increase the training dataset to reduce the over-fitting problem. To show the importance of image augmentation, we have conducted the UNet algorithm training based on diffraction patterns without using any image augmentation techniques. The learning curve between epoch number versus loss and DSC value for UNet algorithm without any image augmentation is given in Fig. 11. It is noted from Fig. 11 that the performance for the training dataset is becoming better and better with the increase of epoch number while the performance for the validation dataset is becoming better to a point and then begins to be worse. This is a typical over-fitting outcome as the algorithm has learned the training dataset well but cannot generalize to new data. The corresponding evaluation results of UNet algorithm based on diffraction patterns from DHM under 40× magnification without image augmentation approach are also given in Table 4. The results have shown that the performance is not as good as these from UNet with image augmentation included during training.

**Table 4:** Evaluation of UNet based on DHM under 40× magnification.

Metrics	RBC hologram (with image augmentation)	RBC phase image (with image augmentation)	RBC hologram (without image augmentation)
DSC	0.91/0.002	0.92/0.003	0.86/0.003
HD	14.88/5.51	12.17/3.34	21.09/4.91
95th HD	4.30/1.24	3.00/0.75	10.21/2.23
Sensitivity	0.96/0.003	0.96/0.002	0.89/0.007
Accuracy	0.99/0.0003	0.99/0.0002	0.92/0.002
Correctly counted cells	323.4/10.03	323.6/8.16	258.5/12.42
Over-counted cells	5.8/2.18	4.3/1.28	14.1/3.57
Ground truth cell number	328/11.84	328/11.84	328/11.84

## 4 Discussion and Conclusions

In this paper, we have trained a deep FCN and used it to do the multiple cell detection and counting from the diffraction pattern directly where the diffraction patterns are recorded from DHM that is a label-free imaging technique. Simulation results have shown that our method can successfully fulfill the purpose of rapidly detecting and counting multiple cells in hologram images at single cell levels. The proposed method reduces the phase reconstruction step in traditional multiple targets detection and counting system, which greatly improves the efficiency and convenience of analysis processing. Furthermore, the proposed deep FCN algorithm is an end-to-end training and prediction technique that can be applied to the whole image and adapt to image with different sizes. Therefore, it can avoid the disadvantages that existed in the CNNs and enable the high-throughput capabilities with a counting rate of more than 288 cells per second and 200  μm×200  μm FOV. Additionally, our system may be further enhanced by larger FOV to increase throughput. In this study, we also tested the deep learning algorithm that was trained using different datasets and obtained different prediction results. Our simulations results reveal that it is better to train and use the model for data obtained at the similar condition. That is, the data used for training and testing should come from the similar distribution. It is highly recommended that the variation of the hologram recording for model inference should be similar to the variation of hologram recording for algorithm training.

For the multiple cell detection and counting from diffraction pattern, our results also indicate that the UNet algorithm can get very good performance when the used hologram images are from DHM under 40× magnification. Compared with the CNN algorithm, the UNet algorithm can get much better results in terms of accuracy and throughput rate. The PSPNet has similar results as UNet and this means other deep FCNs can also work for the multiple cell detection and counting in diffraction pattern from off-axis DHM. The DHM is necessary equipment to obtain the diffraction pattern for the algorithm training and prediction. It may be good to train the model based on high-performance computation system but it is not necessary for the model deployment. Regarding the “black box” feature of deep learning model, some explainable deep learning algorithms can be utilized in the future. We can also apply the explainable machine learning methods, such as decision tree, to the features extracted from deep learning model to do the cell detection and counting. Hence, these experimental results demonstrate that our proposed system can provide a promising tool for rapid cell counting and applications in the field of hematology.
