# Supplementary material for: High-throughput label-free cell detection and counting from diffraction patterns with deep fully convolutional neural networks
Source: J Biomed Opt. 2021 Mar 8;26(3):036001. doi: 10.1117/1.JBO.26.3.036001 (PMC7939515; doi:10.1117/1.JBO.26.3.036001)
Supplement: Supplementary file 1 [file JBO_026_036001_SD001.pdf]

Supplementary Material

High-throughput label-free cell detection and counting from diffraction patterns with deep fully convolutional neural networks

Faliu Yi,[1] Seonghwan Park,[2] and Inkyu Moon[2]
[1]Department of Clinical Science, University of Texas Southwestern Medical Center, Dallas, TX, 73390 USA
[2]Department of Robotics Engineering, Daegu Gyeongbuk Institute of Science & Technology, 333, Techno jungang-daero, Hyeonpung-eup, Dalseong-gun, Daegu, 42988 South Korea

In this supplemental document, we provide tables (Table S1 and Table S2) to include more metric values for the evaluated model. We use figures (Fig. S1 and Fig. S2) to expand the description of concepts such as Hausdorff distance and different types of counted cells. More details about the used deep learning architectures are also given here (Fig. S3 and Fig. S4).

Table S1. Evaluation of model trained on hologram images under 40× magnification

Metrics	RBC hologram(40×)(mean/std)			RBC hologram(20×)(mean/std)			Bead hologram (40×)(mean/std)		
	CNN	UNET	PSPNET	CNN	UNET	PSPNET	CNN	UNET	PSPNET
95th HD	7.57/2.01	4.30/1.24	5.05/1.06	32.03/9.159	29.68/8.27	24.60/3.76	89.81/7.5	79.06/4.49	93.12/10.13
Correctly counted cells	311/13.19	323.4/10.03	318/11.6	218.4/19.49	223.2/16.76	203.2/17.64	113.4/5.43	140.6/18.35	77.4/5.31
Over-counted cells	26.6/1.62	5.8/2.18	6.2/2.8	167.2/12.49	228.2/37.55	150.6/11.74	232.8/9.45	303/31.06	189.4/14.6
Ground truth cell number	328/11.84	328/11.84	328/11.84	294/25.93	294/25.93	294/25.93	334/16.38	334/16.38	334/16.38

Table S2. Evaluation of model trained on hologram images from different source

Metrics	RBC hologram(40×)(mean/std)			RBC hologram(20×)(mean/std)			Bead hologram (40×)(mean/std)		
	CNN	UNET	PSPNET	CNN	UNET	PSPNET	CNN	UNET	PSPNET
95th HD	6.72/1.16	3.07/0.89	6.65/0.41	8.05/4.09	6.15/4.14	5.54/4.67	55.28/7.98	23.57/6.03	32.74/11.31
Correctly counted cells	315/10.25	324/10.20	321.4/11.37	273.8/25.56	292.8/23.76	289.4/24.33	164.2/13.49	243.4/12.17	235.8/11.21
Over-counted cells	23.8/1.72	4.6/1.41	5.4/1.85	13.4/4.84	10.4/4.58	20.8/6.52	60.8/6.04	35.8/9.78	43.6/6.8
Ground truth cell number	328/11.84	328/11.84	328/11.84	294/25.93	294/25.93	294/25.93	334/16.38	334/16.38	334/16.38

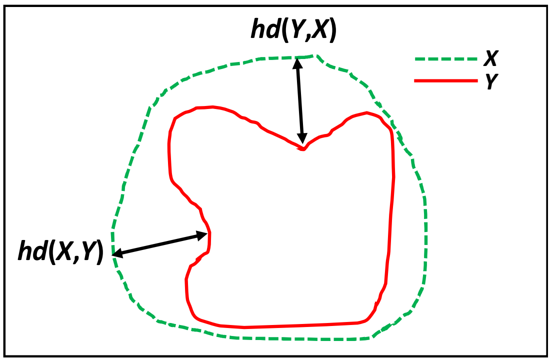

Fig. S1. Illustration of Hausdorff distance between points sets *X* and *Y*.

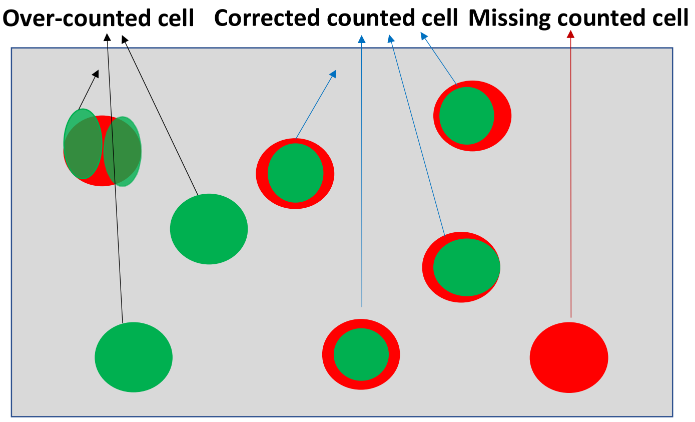

Fig. S2. Illustration of different types of counted cells (red circle denotes ground truth cells; green circle denotes predicted cells).

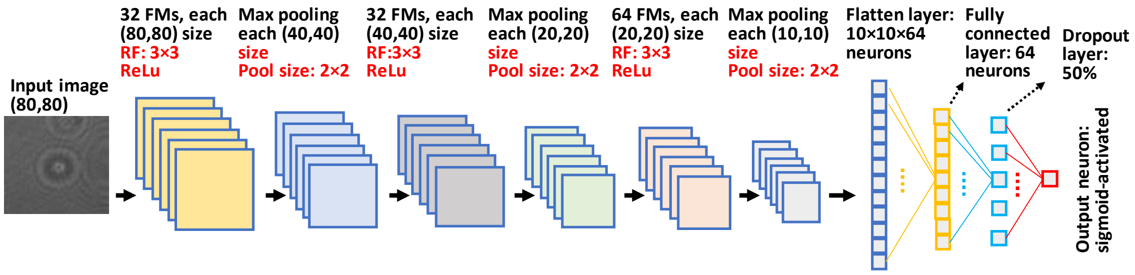

Fig. S3. Illustration of used CNN architecture (FMs: feature map, RF: receptive field).

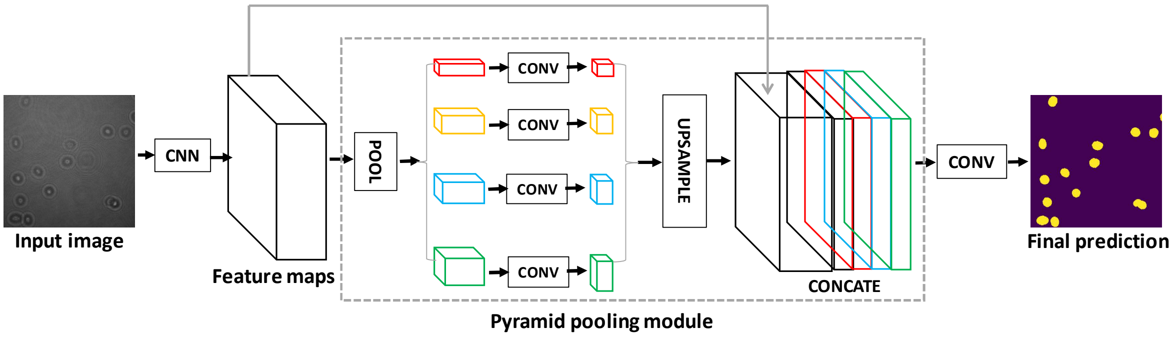

Fig. S4. Illustration of used PSPNet architecture.
